# Stem Cell-Derived Extracellular Vesicles as Potential Therapeutic Approach for Acute Kidney Injury

**DOI:** 10.3389/fimmu.2022.849891

**Published:** 2022-03-10

**Authors:** Marco Quaglia, Guido Merlotti, Andrea Colombatto, Stefania Bruno, Alessandra Stasi, Rossana Franzin, Giuseppe Castellano, Elena Grossini, Vito Fanelli, Vincenzo Cantaluppi

**Affiliations:** ^1^ Nephrology and Kidney Transplantation Unit, “Maggiore della Carità” University Hospital, Department of Translational Medicine, Translational Research on Autoimmune and Allergic Disease (CAAD), University of Piemonte Orientale (UPO), Novara, Italy; ^2^ Department of Medical Sciences, University of Torino, Torino, Italy; ^3^ Nephrology, Dialysis and Transplantation Unit, Department of Emergency and Organ Transplantation, University of Bari, Bari, Italy; ^4^ Nephrology, Dialysis and Kidney Transplantation Unit, Fondazione IRCCS Ca’ Granda Ospedale Maggiore Policlinico, University of Milan, Milan, Italy; ^5^ Laboratory of Physiology, Department of Translational Medicine, Translational Research on Autoimmune and Allergic Disease (CAAD), University of Piemonte Orientale, Novara, Italy; ^6^ Department of Anesthesiology and Intensive Care, University of Torino, Torino, Italy

**Keywords:** acute kidney injury, acute tubular necrosis, ischemia-reperfusion injury, sepsis, chronic kidney disease, stem cell, extracellular vesicles, regenerative medicine

## Abstract

Acute kidney injury is a frequent complication of hospitalized patients and significantly increases morbidity and mortality, worsening costs and length of hospital stay. Despite this impact on healthcare system, treatment still remains only supportive (dialysis). Stem cell-derived extracellular vesicles are a promising option as they recapitulate stem cells properties, overcoming safety issues related to risks or rejection or aberrant differentiation. A growing body of evidence based on pre-clinical studies suggests that extracellular vesicles may be effective to treat acute kidney injury and to limit fibrosis through direct interference with pathogenic mechanisms of vascular and tubular epithelial cell damage. We herein analyze the state-of-the-art knowledge of therapeutic approaches with stem cell-derived extracellular vesicles for different forms of acute kidney injury (toxic, ischemic or septic) dissecting their cytoprotective, regenerative and immunomodulatory properties. We also analyze the potential impact of extracellular vesicles on the mechanisms of transition from acute kidney injury to chronic kidney disease, with a focus on the pivotal role of the inhibition of complement cascade in this setting. Despite some technical limits, nowadays the development of therapies based on stem cell-derived extracellular vesicles holds promise as a new frontier to limit acute kidney injury onset and progression.

## Introduction: Definition and Classification of Acute Kidney Injury

**Figure 1:**
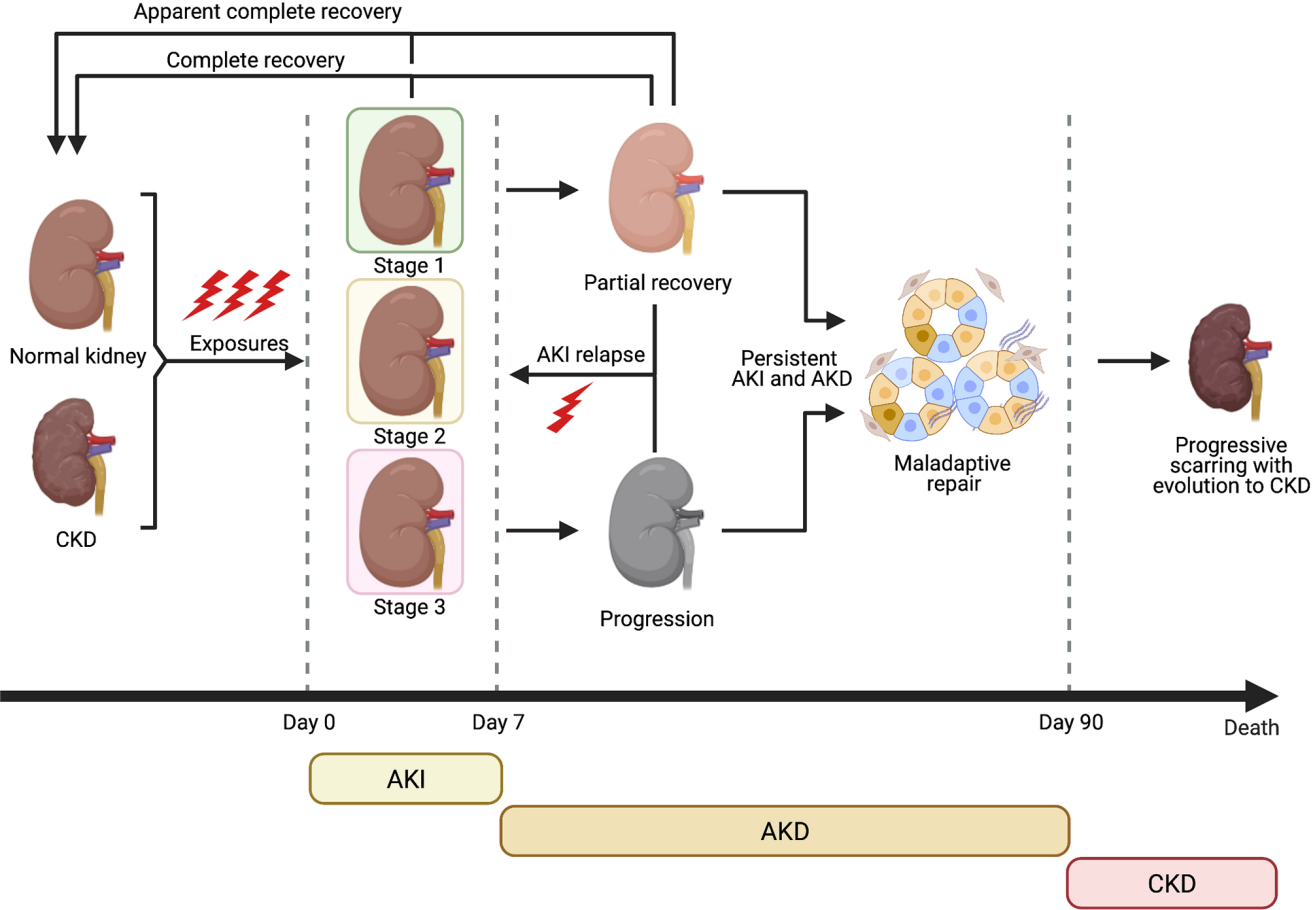
Clinical course of AKI and evolution towards CKD (Created with BioRender.com).

The term Acute kidney injury (AKI) indicates a sudden worsening of renal function due to acute renal damage, with consequent accumulation of nitrogenous waste products and alteration of hydrosaline and acid-base homeostasis. In the past decades, several criteria have been proposed in order to uniform the definition of AKI: the recommendation statements of 2012 Kidney Disease Improving Global Outcomes (KDIGO) Clinical Practice Guideline define AKI as a rise in the serum creatinine (sCr) level by 0.3 mg/dl within 48 hours, or a 1.5-fold increase from baseline within prior 7 days; or oliguria (<0.5 ml/kg/h for 6 hours). In addition, AKI is staged for severity – from mild stage 1 to most severe stage 3 – according to serum creatinine values and urine output, usually classifying patients requiring renal replacement therapy (RRT) as stage 3 KDIGO. Several causes may induce AKI in patients with or without underlying chronic kidney disease (CKD); a potential classification is based on pathophysiological mechanisms of renal injury such as kidney hypoperfusion (pre-renal AKI), parenchymal kidney diseases (intra-renal or parenchymal AKI, which includes acute tubular necrosis, ATN) and obstruction of the urinary tract (post-renal AKI). Incomplete recovery of an AKI event due to persistence of renal pathophysiologic process can lead to AKI-CKD transition, especially in patient with some degree of pre-existent CKD (**Figure 1**). As for AKI epidemiology, incidence and prevalence are not well defined due to different AKI definitions. Despite these limitations, a metanalysis of Susantitaphong et al. (1) observed that in-hospital AKI incidence was 22% – using 2012 KDIGO AKI definition – while it reached 57% in intensive care units (ICU) according to the multinational AKI-EPI study (2). Overall AKI incidence seems to be rising in the United States and it is associated with higher health care costs, greater long-term care, increased risk of CKD and hospital mortality (3, 4). This increase especially affects Afro-American population, due to genetic factors which also condition a reduced number of nephrons. A large metanalysis (5) and recent studies (6) confirmed that Black race is an independent risk factor for AKI.

Similar to CKD, other factors associated to the rise of AKI incidence are older age, increasing burden of comorbidities (e.g. hypertension, diabetes mellitus, CKD, heart failure, sepsis, cancer), improved clinician’s awareness (leading to inclusion of less severe forms), growing use of nephrotoxic drugs and increasing frequency of surgical and angiographic procedures (7, 8). Among these, older age is strongly associated with AKI incidence through multifactorial mechanisms. Renal senescence reduces nephron number and functional reserve, predisposing to relapsing AKI episodes and also to maladaptive repair, incomplete recovery and AKI-CKD transition. This process can be considered as a form of accelerated renal senescence and will be analysed in a specific Section (9, 10).

Different causes related to ischemia-reperfusion injury due to hypovolemia and/or hypoperfusion, administration of nephrotoxic drugs and the presence of sepsis/septic shock have been identified as hallmarks for AKI development. Recent studies have also provided new insights into complex AKI pathophysiology, with remarkable progress especially in the field of sepsis-associated AKI (s-AKI).

Sepsis is a dysregulated immune response to infection that causes multiple organ dysfunction: the immune response after a septic insult is characterized by unbalanced hyperinflammation and immune suppression (11). Sepsis-associated excessive inflammation is sustained by several cell types including leukocytes (neutrophils, macrophages, natural killer cells), endothelial cells (EC), cytokines, complement products, and the coagulation system (12). The widely used concept of “cytokine storm” refers to the release of proinflammatory cytokines such as TNF-alpha, IL-1b, IL-12, and IL-18 that may contribute to organ injury (12). However, a complex network of several mediators is embedded in sepsis-associated multiple organ dysfunction such as the release of extracellular traps by neutrophils (NETs) (13), the complement activation resulting in the release of the anaphylatoxins C3a and C5a, the immunothrombosis in the microvasculature mainly triggered by tissue factor that initiates blood coagulation by forming a complex with clotting factor (F) VIIa, thereby inciting blood coagulation by activating FX and FIX (14). These alterations have been also recently described in COVID-19-associated AKI (15).

Sepsis represents nowadays the most frequent cause of AKI in critically ill patients admitted to Intensive Care Units (ICU) with pathogenic mechanisms similar to those described above for immune dysfunction. Indeed, experimental and clinical studies aimed to evaluate kidney perfusion clearly demonstrated that during sepsis, AKI can develop in the presence of a normal or even increased renal blood flow: this finding suggested the presence of other key mechanisms of tissue injury such as microvascular derangement, endothelial dysfunction, inflammation, metabolic reprogramming and sub-lethal injury of tubular epithelial cells, overthrowing the simplistic old paradigm based on kidney hypoperfusion (16, 17). Recent works have further shed light on these etiopathogenetic mechanisms: for example, interaction of damage-associated molecular pattern (DAMPs) and pathogen- associated molecular pattern (PAMPs) molecules with Toll-like Receptors (TLRs) expressed on renal tubular epithelial cells (RTEC) and other renal resident cells can activate several pro-inflammatory pathways (18) and mitochondrial DNA (mDNA) damage due to oxidative stress also plays a key role in inducing RTEC dysfunction (19). Basically, PAMPs and DAMPs can be freely filtered by glomeruli, a process favored by sepsis-induced increased permeability, thus reaching the tubular lumen and inducing functional alterations of RTECs; moreover, microvascular damage of ECs located in the peritubular capillaries may enhance metabolic alterations of RTECs (20).

Innate and adaptive immunity activation during AKI have been increasingly recognized to play an important role in AKI. Cross-talk between RTECs and immune cells through a network of cytokines seems to be essential to induce T cell phenotype changes which affect AKI evolution (21). In this setting an important role of IL-15 has been delineated. RTECs express IL 15 and its receptors and intrarenal IL 15 levels inversely correlate with AKI severity in experimental models. Inhibition of RTECs apoptosis during pathological stress appears to explain IL 15 protective effects (22) Furthermore, IL 15 can reduce extracellular matrix (ECM) synthesis by myofibroblasts and monocyte chemoattractant protein (MCP-1) release by macrophages and also inhibits Transforming Growth Factor-β (TGF-β) 1-induced RTEC epithelial-mesenchymal transition (EMT): these multiple anti-fibrotic effects suggest a role in the prevention of AKI-CKD transition (23, 24).

Despite progress in understanding AKI pathophysiology, however, efforts to develop targeted therapies has not led to robust results yet (16) and treatment is still mainly supportive (e.g. hemodynamic stabilization, dose adjustment or discontinuation of nephrotoxic drugs, antibiotic therapy in s-AKI) (25–28). Also in s-AKI, neither pharmacological approaches nor extracorporeal blood purification therapies aimed at removing PAMPs and DAMPs have led to a significant improvement of in-hospital AKI incidence and mortality (16).

In this setting, stem-cell (SC) therapy and SC-derived extracellular vesicles (EVs) represents a new frontier in the treatment of acute and chronic kidney disorders, because of their anti-inflammatory, immunomodulatory and regenerative properties. Recent studies observed the beneficial therapeutic properties of Mesenchymal Stromal Cells (MSCs) in ischemic AKI, renal transplantation, lupus nephritis and diabetic nephropathy (29, 30). These actions are mainly paracrine and mostly mediated by transfer of EVs containing microRNAs, mRNAs, and proteins that reprogram cell functions *via* immunomodulatory and regenerative effects (31), as detailed in the following section.

In this review, we summarize the state-of-the-art knowledge on EVs derived from different types of stem cells (SCs) as therapy of AKI, focusing on impact of EVs on different pathophysiological mechanisms underlying toxic, ischemic and s-AKI (29–31) and AKI-CKD transition.

## General Features of Stem Cells and Extracellular Vesicles (EVs)

SCs are unspecialized cells with self-renewal capacity, which can potentially differentiate into any cell type of organism. Their therapeutic potential is revolutionizing regenerative medicine and is providing promising applications also in the field of Nephrology (32).

Among progenitor cells, MSCs represent the most studied type over the last decades: MSCs are adult multipotent stromal cells with a high proliferative potential, derived from non-hematopoietic precursors. They can differentiate into mesenchymal (osteocytes, adipocytes and chondroblasts) and non-mesenchymal lineages (33). Initially found in bone marrow (BM) (34), they were subsequently isolated from multiple fetal and adult tissues such as adipose tissue (AD-MSCs), umbilical cord blood (UC-MSCs), fetal membrane (FM-MSCs) and human placenta (hP-MSCs) (35–37). This makes them one of the most accessible SC type and an attractive candidate source to develop products for cell therapies (38). Due to the ease of preparation, MSCs remain the most common option among cellular therapies and have already proven to be safe and effective in reducing AKI in experimental models and clinical trials, displaying cytoprotective, regenerative and immunomodulatory properties (39, 40).

Other types of SCs have been recently investigated as a therapy for AKI, including inducible Pluripotent Stem Cells (iPSCs). These are derived from differentiated adult cells (e.g. keratinocytes, fibroblasts) which are induced into pluripotency by exposing them to specific reprogramming factors through viral vectors and directed towards renal lineages such as podocyte progenitors (41). Administration of iPSC has proved to be effective in a rat model of AKI, reducing oxidative stress and inflammation (42).

Spermatogonial stem cells (SCCs) have also been shown to be capable of differentiating into pluripotent stem cell lines, converting into embryonic-like SCs and differentiating into renal tubular-like cells. Also, this type of SC has shown promise in restoring kidney function after AKI (43).

Endothelial progenitor cells (EPCs), a BM-derived progenitor type able to circulate in the bloodstream, play a major role in vascular integrity by protecting ECs and promoting angiogenesis and recovery also in AKI models (44).

Of interest, the beneficial effects of all these SC types are predominantly mediated by paracrine/endocrine actions, *via* secretion of growth factors and especially EVs. The latter represent crucial components of cellular secretome and mediate intercellular communication through transfer of bioactive molecules between originator and recipient cells, especially mRNAs and microRNAs (miRNAs), modifying phenotype and function of target cells (45, 46). As their parental cells, EVs are not immunogenic and may successfully activate regenerative processes in injured cells and tissues (47, 48).

Recently, EVs released from renal cells themselves have been investigated as therapy for AKI: kidney-derived MSCs (49), glomerular and tubular renal progenitors (50), renal tubular epithelial cells (RTEC) (51) and even urinary EVs (uEVs) from healthy subjects have shown initial promising results in this setting (52). The efficacy of SC-derived EVs has been demonstrated in different settings including numerous studies showing an interesting cross-kingdom communication: EVs from different eukaryotic and prokaryotic kingdoms are involved in many processes including host-pathogen interactions and modulation of cellular functions (53). Of note, some studies showed that exogenous dietary RNAs of plant and animal origin are protected from food processing and gut microenvironment through encapsulation within EVs. EV-carried RNAs (in particular miRNAs) are able to exert biological activities between the host and gut microbiota influencing organ function in the recipient after ingestion (54). Our group has already demonstrated the horizontal transfer of mRNAs and miRNAs from SC-derived EVs of human origin in rat cells: indeed, in a rat model of anti-Thy1.1-induced mesangioproliferative glomerulonephritis, EVs released from human Endothelial Progenitor Cells (EPCs) horizontally transferred to rat mesangial cells distinct mRNAs coding for Factor H, CD55 and CD59, thus inhibiting complement-induced apoptosis and C5b-9/C3 mesangial cell deposition (55). Moreover, the same type of EVs protected the kidney from ischemia-reperfusion injury in rats by delivering their RNA content, the miRNA cargo of which was shown to contribute to reprogramming hypoxic renal endothelial and tubular epithelial cells to a regenerative program (56).

EV-based therapeutic approach has some advantages compared to cell-therapy. First, EVs exhibit a superior efficacy profile as they pass through the blood-tissue barriers and efficiently reach injured cells (57). Second, no adverse immune responses have been reported in patients undergoing allogeneic administration of SC-EVs and no evidence of oncogenic potential of SC-EVs has been reported. In fact, they can inhibit tumor growth by interfering with cell cycle and inducing apoptosis and/or necrosis of cancer cells (58, 59). Thus, EVs represent a feasible, cell-free therapeutic alternative and their role has recently been investigated in several renal diseases (45, 46), especially in AKI. This is the setting in which therapeutic properties of SC-EVs and the rationale for their employment has been better defined and represents the main focus of this Review.

## Rationale of Therapy With EVs in AKI

**Figure 2:**
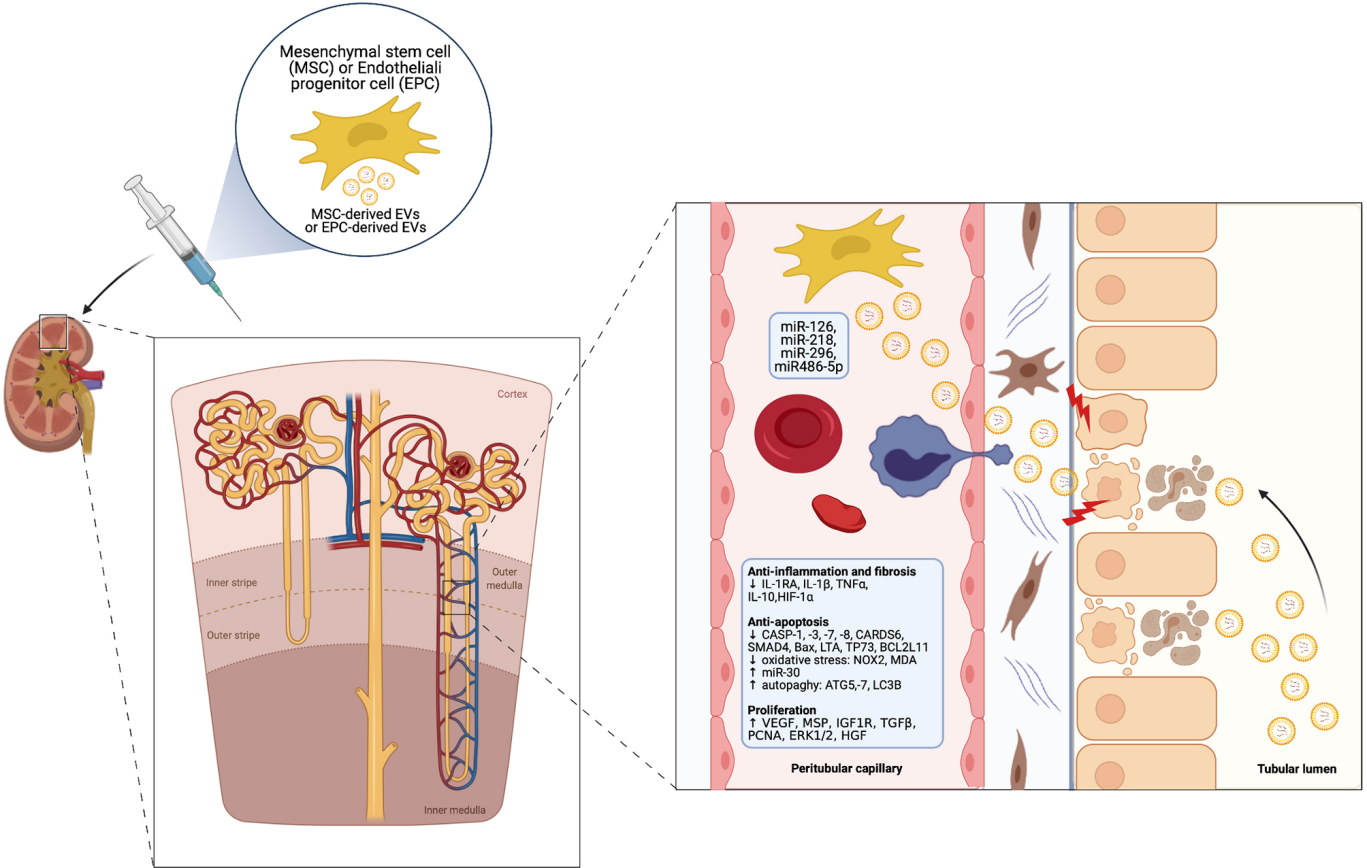
Cytoprotective, regenerative and immunomodulatory effects of MSC-derived EVs in the setting of AKI (Created with BioRender.com).

A growing body of pre-clinical studies indicates a potential therapeutic role of EVs derived from MSCs and other progenitor cell types especially in IRI-induced AKI. EVs can shuttle miRNAs and other genetic material into injured renal cells – such as RTEC and ECs – and epigenetically re-programme them. This leads to activation of signaling pathways, which exerts multiple beneficial effects within three main areas (60, 61) (**Figure 2**).

- renal protection: inhibition of apoptosis/necrosis, oxidative stress, senescence and fibrogenesis; promotion of autophagy (62).
- renal regeneration: stimulation of cell proliferation, migration, tubular dedifferentiation, angiogenesis (63).
- immunomodulation: anti-inflammatory and immunosuppressive effects, mainly through induction of M2 macrophages and T-regulatory cells (Treg) (64) and modulation of NK cell activity (65).

The combination of these effects can heal injured RTECs and ECs, thus promoting regression of AKI (66). Of note, pre-treatment with RNAase consistently abolished these effects, indicating that mRNA and miRNA transfer from SCs to injured renal cells is crucial in this healing process (67). Ferguson et al. identified 23 top-miRNAs which account for over 79% of total miRNAs in MSC-derived exosomes and seem to mediate the predominant effects, targeting 5481 genes (68). A comprehensive review of miRNA repertoire carried by SC-derived EVs for AKI treatment has been recently published and it is beyond the scope of this work (69).

## Therapy With SC-Derived EVs in Different Forms of AKI

As previously mentioned, while most studies were performed with EVs derived from BM-MSCs, UC-MSCs and AD-MSC, other EV sources have been recently employed, including kidney resident populations (50, 70) and uEVs (71). Potential mechanisms of actions and main mediators, with a focus on miRNAs, will be briefly analyzed. A summary of the main studies on EVs as therapy of AKI is outlined in **Table 1**, in which they are categorised according to type of originating cell type (49–51, 56, 65–67, 72–96).

**Table 1:** EVs derived from MSCs or other cell types as therapy of AKI.

Source of EVs	Type of experiments	Mechanisms of action and mediators	References
Human BM-MSC	Animal model (mice) *In-vitro*	Increased proliferation of RTECs	(66)
Human Liver Stem cells (HLSC)	Animal model (mice) *In-vitro*	Increased proliferation and reduced apoptosis of RTECs	(72)
Human BM-MSC	Animal model (mice) *In-vitro*	Increased proliferation and reduced apoptosis of RTECs; anti-inflammation (upregulation of genes involved in metabolic pathways and downregulation of genes involved in inflammation)	(73)
Human UC-MSC	Animal model (rat) *In-vitro*	Increased proliferation and reduced apoptosis of RTECs	(74)
Human BM-MSC	Animal model (rat)	Increased proliferation and reduced apoptosis of RTECs; protection against chronic kidney injury	(67)
Human BM-MSC	*In-vitro*	Reduced apoptosis (EVs transfer miR-148b-3p, miR-410, miR-495, miR-548c-5p and miR-886-3p to RTECs)	(75)
Human UC-MSC	Animal model (rat) *In-vitro*	RTECs dedifferentiation and proliferation (increased ERK1/2 and HGF expression)	(76)
BM-MSC	Animal model (mice) *In-vitro*	Reduced RTEC apoptosis (inhibited NLRP3 expression through miR-223)	(77)
Mouse kidney resident glomerular progenitors (Gl-MSC)	Animal model (mice) *In-vitro*	Increased RTECs proliferation	(50)
Human UC-MSC	Animal model (rat) *In-vitro*	Increased RTECs proliferation (releasing from G2/M cell cycle arrest).	(78)
Human BM-MSC	Animal model (mice) *In-vitro*	Inhibition of RTECs apoptosis (downregulation of Sema3A expression and activation of AKT/ERK pathways through miR-199a-3p); inhibition of NK.	(79)
Rat BM-MSC	Animal model (rat) *In-vitro*	Anti-inflammation (reduced IL1β and TNFα)	(80)
Human BM-MSC	Animal model (rat) *In-vitro*	Immunosuppression (NK cells inhibition)	(65)
Human UC-MSC	Animal model (rat) *In-vitro*	Anti-oxidation (decreased expression of NOX2 and activation of Nrf2/ARE)	(81, 82)
Human UC-MSC	Animal model (rat)	Inhibition of mitochondrial fission (miR-30) and reduced RTECs apoptosis	(83)
Human BM-MSC	Animal model (mice) *In-vitro*	Suppression of ER stress (through miR-199a-5p)	(84)
Mouse kidney resident MSC	Animal model (mice) *In-vitro*	Increased angiogenesis; increased proliferation and reduced apoptosis	(49)
EPC	Animal model (rat) *In-vitro*	Increased angiogenesis	(56)
ECFC	Animal model (mice) *In-vitro*	Increased angiogenesis (transfer of miR-486-5p to EC inhibits apoptosis and Endo-MT)	(85, 86)
RAVPCs	Animal model (mice) *In-vitro*	Increased angiogenesis, increased ECs migration (transfer of miR-218)	(87)
Human BM-MSC	*In-vitro*	Increased angiogenesis (transfer of miR-125a)	(88)
MSC-EVs	Animal model (rat) *In-vitro*	Increased angiogenesis	(89)
Human BM-MSC	Animal model (mice) *In-vitro*	Increased angiogenesis (transfer of miR-199a-3p)	(79)
Human UC-MSC	Animal model (rat) *In-vitro*	Anti-inflammation (downregulation of CX3CL1, reduction in CD68+ macrophages infiltration) and decreased renal fibrosis (reduction of α-SMA). Downregulated CX3CL1 was associated with specific miRNAs in EVs (miR-15a, miR-15b, mi-R16).	(90)
BM-MSCs	Animal model (mice) *In-vitro*	Anti-inflammation (EVs enriched in CCR-2 suppress macrophage functions)	(91)
Rat AD-MSC	Animal model (rat)	Anti-oxidation, inhibition of apoptosis and renal fibrosis	(92)
Human UC-MSC	Animal model (rat)	Inhibition of apoptosis, increased proliferation of RTECs; Anti-inflammation (reduced CD68+macrophages infiltration); Anti-fibrosis (decreased expression of α-SMA and TGFβ; Increased expression of HGF)	(93)
HPC-Human RTECs	Animal model (rat) *In-vitro*	Hypoxia can enhance and differentiate EVs regenerative properties compared with EVs released under normal oxygenation. HPC-Human RTECs can release EVs with anti-oxidant properties and the potential to induce SCs differentiation normal RTEC	(51, 94)
AD-MSC	Animal model (mice) *In-vitro*	Increased proliferation of RTECs; Inhibition of AKI-to-CKD transition (activation of Sox 9)	(95, 96)

EC, Endothelial Cells; ER, endoplasmatic reticulum; HLSC, Human Liver Stem Cell; HGF, Hepatocyte Growth Factor; ECFC, Endothelial Colony-forming Cell; EPC, Endothelial Progenitor Cells; RAVPC, Renal Artery Vascular Progenitor Cells; Gl-MSC, Glomerular progenitor MSC; NLRP3, NLR family-pyrin domain containing 3.

### Nephrotoxic AKI

Several cellular sources of SCs have been tested in models of toxic AKI. Bruno et al. first demonstrated that BM-MSC-derived EVs facilitated morphological and functional renal recovery in a model of glycerol-induced AKI mimicking rhabdomyolysis-associated tubular damage (66). Human liver stem cells (HLSCs) cells were then tested in the same experimental model with similar results. A single intravenous injection of HLSCs facilitated histological and functional renal recovery through the induction of RTEC proliferation and inhibition of apoptosis. Of interest, EVs were the main component of HLSC-derived conditioned medium capable of promoting regeneration (72).

Furthermore, uEVs have been recently employed in the same mouse model of glycerol-induced toxic AKI. Intravenously injected uEVs stimulated RTEC proliferation, reduced expression of inflammatory markers and restored endogenous Klotho. Interestingly, murine uEVs derived from Klotho-null mice lost these reno-protective effects, suggesting a key-role of Klotho in mediating uEVs beneficial effects on RTECs (52).

EVs obtained from BM-MSCs were also tested in a lethal cisplatin-induced AKI model, in which they stimulated RTEC proliferation and conferred them resistance to apoptosis *in vitro*, resulting in improvement of renal function and morphology in an *in vivo* SCID mouse model (73). Similar results were reported employing UC-MSCs-derived EVs in the same experimental setting (74).

Last, BM-derived MSC repaired but did not prevent gentamycin-induced AKI (97).

### Ischemic AKI

SC-derived EVs have also been studied in several models of ischemia/reperfusion injury (IRI)-associated AKI, as outlined in **Table 1**. In all of these experimental models, administration of EVs derived from different cell types after IRI accelerated recovery of renal function and/or decreased histological tubular injury through multiple mechanisms (61).

Gatti S et al. demonstrated a beneficial effect of BM-MSC-derived EVs in favouring recovery from ischemic AKI and the need for multiple injections to achieve renal function normalization. This effect was mainly due to upregulation of anti-apoptotic genes in injured RETCs and prevented transition from AKI to CKD (67).

Of interest is the fact that i.v. administered human MSC-derived EVs were effective in alleviating renal damage even in rats which had received kidney transplant after cardiac death, a setting characterized by severe IRI (93).

Another study highlighted the role of specific miRNAs within the cargo of MSC-derived EVs (miR-148b-3p, miR-410, miR-495, miR-548c-5p, miR-886-3p) in protecting RTECs from IRI in an *in vitro* model induced by ATP-depletion. Down-regulation of miRNAs involved in apoptosis, hypoxia and cytoskeletal reorganization mediated this effect (75).

Other studies brought out a wide spectrum of beneficial actions of BM-MSC-derived EVs in ischemic AKI: they can induce RTEC dedifferentiation and growth *via* hepatocyte growth factor (HGF) induction (76) and prevent apoptosis through transfer of miR-21 (98) and miR-223 (77); they inhibit CXC3CL1, blunting evolution towards fibrosis (90)**;** finally, they are enriched in chemokine receptor type 2 (CCR-2), which enables them to buffer extracellular free chemokine ligand 2 (CCL-2), suppressing macrophage functions (91).

Of note, several EVs biological activities are specifically related to aerobic metabolism; for example, MSC-derived EVs can carry respiratory complexes, supporting an independent aerobic metabolism when mitochondrial respiratory capacity is impaired (99); they can inhibit mitochondrial fission through miR-30 transfer (83) and re-establish adequate intracellular ATP levels, with beneficial epigenetic changes such as reversion of histone H2 and H2B up-regulation, a typical feature of apoptotic cells (100); they can attenuate mitochondrial damage in RTECs by stabilising mitochondrial DNA (mDNA) previously depleted by oxidative stress (101).

Furthermore, they might contribute to the anti-oxidant potential of injured cells, for example down-regulating calnexin, a nicotinamide adenine dinucleotide phosphate (NADPH)-oxidase 4 (NOX4)-interacting protein (102).

Similarly, Zhang et al. reported that human Warton Jelly (hWG) MSC-derived EVs can reduce expression of NADPH-oxidase 2 (NOX2) and stimulate the nuclear factor eryhroid 2-related factor 2 (NRF-2) and anti-oxidant response element pathway (81, 82). All these actions result in an overall reduction of reactive oxygen species (ROS) formation and could limit tubular cell death and senescence after re-oxygenation.

EVs derived from iPSC (103) have also been shown to protect mitochondrial function and regulate several genes associated with oxidative stress. Interestingly, iPSC-EVs showed a higher efficiency in renal protection than AD-EVs (104, 105).

Another aspect which contributes to reparative effects of EVs is their capacity to promote angiogenesis, counteracting renal hypoxia. EPCs administration determined increased tubular proliferation and reduction in capillary rarefaction, glomerulosclerosis and tubulointerstitial fibrosis in a rat model of IRI-associated AKI, suggesting protection against post-IRI fibrosis. In this study, pro-angiogenic miR-126 and miR-296 shuttled by EPC-derived EVs to ECs located in peritubular and glomerular capillaries accounted for this effect, as treatment with RNAase or specific antagomiRs abolished it (56). Other studies subsequently proved that similar pro-angiogenetic effects were mediated by EVs released from other human SC types, such as kidney-derived MSCs (49, 50), endothelial colony-forming cells (ECFCs) (85, 86), vascular progenitor cells derived from renal arteries (87) and BM-MSCs (79, 88, 89).

Interestingly, the study of Zou X et al. showed that hWJ-MSC-derived EVs upregulated vascular endothelial growth factor (VEGF) and downregulated hypoxia-inducible factor 1 (HIF-1α) in a rat model of IRI and that VEGF was through directly transferred by EVs (89).

Recent studies have shown that hypoxia preconditioning (HPC) can improve and differentiate EVs regenerative properties compared with EVs released under normal oxygenation, as secreted EVs convey the metabolic state of originating cell, including trophic factors protecting against hypoxia (106).

In the study by Collino et al. peculiar anti-apoptotic, anti-oxidative, mitochondrial energy-supply and pro-angiogenic pathways were activated by hypoxic AD-MSC derived EVs and induced a distinct proteomic pattern is in RTECs (107). Four effects were specifically enhanced in hypoxic EVs: downregulation of fibroblast growth factor receptor 1 (FGFR-1) and reduction of Transforming Growth Factor β-1 (TGFβ-1)-induced epithelial-to-mesenchimal transition (EMT) (108); promotion of angiogenesis through vascular endothelial growth factor (VEGF), blunting renal microvasculature rarefaction (109); translocation of Nrf-2 into the nucleus, activating antioxidant genes including Heme-oxigenase-1 (HO-1) (110); downregulation of IL6, reducing macrophage infiltration and polarization towards a M2 phenotype (111).

Hypoxic conditions have also been shown to promote angiogenic potential of iPSC-derived EVs (112).

An interesting aspect is that injured RTECs can themselves, especially when treated with HPC (113, 114), release EVs with anti-oxidant properties (94) and have the potential to modulate phenotypic and functional features of SCs, stimulating them to differentiate into normal RTEC. This effect may be ascribed to the release of a specific EV phenotype by de-differentiated tubular cells (57).

In another study, Dominguez JM et al. harvested hypoxic human RTECs and derived EVs from kidneys declined for transplantation and demonstrated that, after injecting them into nude rats exposed to bilateral renal ischemia, they both preserved renal function; however, EVs proved superior in maintaining renal vascular and epithelial networks, preventing oxidative stress and blunting pro-inflammatory and fibrogenic pathways. Proteomic analysis demonstrated broad ischemia-induced alterations at all cell levels and prevention of major drift in transcriptome by EV infusion (377 out of 628 altered proteins were “corrected” by EVs). This resulted in a reduced risk of evolution towards fibrosis and CKD (51).

On this basis, association of normoxic and hypoxic EVs has been proposed with the rationale of integrating respective peculiar effects (92, 115).

Finally, a recent study by Liu et al. showed that encapsulation of EVs isolated from human placenta (hP)-derived MSCs in a collagen matrix improved their retention in an AKI murine model, remarkably enhancing their therapeutic effects (inhibition of RTEC proliferation and of endoplasmic reticulum stress, stimulation of angiogenesis) compared with hP-MSC-derived EVs alone (116).

Taken together, all these studies indicate that SC-derived EVs have a multi-level therapeutic potential and that different type of EVs may specifically target pathophysiological aspects involved in ischemic AKI, either preventing it or accelerating its recovery.

### Sepsis-Associated AKI (s-AKI)

As described above, sepsis is a common and life-threatening systemic disorder often leading to AKI in a clinical scenario of multiple organ failure due to the maladaptive host response to infection. S-AKI is not merely a consequence of ischemic damage due to hypoperfusion (renal overflow is in fact often normal or even increased) but recognizes a more complex pathogenesis. This includes microvascular damage and intrarenal redistribution of renal blood flow, activation of immune cells and complement with massive release of inflammatory molecules causing RTEC dysfunction (autophagy and mitophagy; arrest of cell cycle; dedifferentiation), endocrine dysregulation (16).

Transfer of miRNAs, mRNAs and proteins from activated immune and ECs through EVs may play a pivotal pathogenetic role in these processes but, on the other hand, it may also represent a therapeutic option for the use of SC-derived EVs (117). For example, EPC-derived EVs carrying mi-RNA-93 5p conferred renal protection in a LPS-induced mouse model of S-AKI, also alleviating multiple organ injury and vascular leakage (118) and blunted LPS-induced HK2 cell injury in another study (119).

During infections, MSC-derived EVs have been shown to eliminate pathogens and to regulate immune response through the secretion of antimicrobial factors, both inhibiting the replication of pathogens and activating the phagocytic function of macrophages (120). In a mouse model in which Escherichia Coli-derived outer membrane vesicles were intraperitoneally injected to establish sepsis, MSC-derived EVs significantly suppressed cytokine release into the systemic circulation, as well as PMN and monocyte infiltration in the peritoneum, by upregulating IL-10 production (121). In experimental models of sepsis obtained by LPS administration or by cecal ligation and puncture (CLP) MSC-derived EVs inhibited the development of disease by downregulating JMJD3 and inactivating the NF-κB signaling pathway through the selected transfer of miR-27b (122). EVs isolated from AD-MSCs have been shown to attenuate inflammation and protect from organ dysfunction by regulating the Notch-miR148a-3p signaling axis and decreasing macrophage polarization to M1 (123). In experimental s-AKI, the administration of EVs isolated from AD-MSCs exerted a renal protective effect through SIRT1 signaling pathway (124). In another CLP model of s-AKI, EVs obtained from UC-MSCs decreased IRAK1 expression through the up-regulation of miR-146b level, inhibited NF-κB activity and limited AKI and mortality (125). These results suggest an important immunomodulatory effect induced by MSC-EV administration in sepsis and a specific protective effect from AKI.

Last, EVs derived from mice pre-treated with remote ischemic preconditioning, elicited by brief periods of IRI in femoral arteries, appears to protect against s-AKI through miR-21, which integrates into RTECs and targets the downstream PDCD4/NF-κB and PTEN/AKT pathway (126).

## The Process of AKI toCKD Transition

Recent studies have demonstrated that maladaptive repair after an AKI episode can predispose to evolution towards CKD and end-stage renal disease (ESRD) (127–129). Different factors appear to contribute to maladaptive repair during AKI, including oxidative stress, DNA damage, microvascular rarefaction, tubular loss, early fibrosis induced by endothelial-to-mesenchymal transition (EndMT) and pericyte-to-mesenchymal transition (PMT), lymphocyte infiltrates, inflammatory cytokine storm (128, 129). Pathophysiology of AKI-CKD transition has been the focus of research and complement system activation is increasingly recognised as having an essential role in this inflammatory scenario, which is closely related to renal senescence (130). These aspects and the potential of EVs as a tool to treat AKI and prevent AKI-CKD transition will be analysed in the following sections.

### The Role of Complement in Renal Senescence and AKI-CKD Transition

In addition to liver synthesis, complement components can derive from renal cells (131) and complement C3 can be expressed by proximal RTECs, ECs, glomerular epithelial and mesangial cells after IRI (132, 133). Recent evidence indicates a crucial role of complement activation in renal tubules and vessels in AKI secondary to rhabdomyolysis (134) and trauma (135).

An aberrant complement activation and a high number of senescent cells also characterise both renal senescence and AKI-CKD transition, which is currently viewed as an accelerated form of kidney aging (136–138) and shares the same pathway and intracellular mediators (139, 140). Thus, there is actually a tight relationship between AKI itself and mechanism of senescence activation (129). From a molecular point of view, senescence refers to a well-defined program associated with cell cycle arrest, apoptosis inhibition and a pro-inflammatory “senescence-associated secretory phenotype” (SASP) (141). The SASP secretome relies on the production of a wide range of pro-inflammatory cytokines, chemokines, growth factors and matrix degrading factors promoting spread of senescence and fibrosis (142). Chronic accumulation of SASP cells leads to “inflammaging”, a persistent, low-grade inflammatory state which causes tissue deterioration (143, 144). Renal senescent cells can be detected by several markers, including loss of key nephroprotective factors such as Klotho. This transmembrane protein, expressed mainly on proximal RTECs where it interacts with the fibroblast growth factor receptor (FGF-23), regulates phosphate homeostasis (145) and exerts anti-fibrotic and anti-inflammatory actions through its 65-kDa soluble form, released into the bloodstream and urine (146). Disruption of Klotho gene determines shortened life span due to premature arteriosclerosis in mice (147) and AKI-induced Klotho deficiency accelerates renal fibrogenesis, retards renal tissue regeneration and promotes AKI-CKD transition (148, 149). Aberrant complement activation during AKI triggers inflammaging and represents an important link between AKI and CKD. Complement activation is involved in two key processes leading to CKD lesions: endothelial-to-mesenchymal transition (EndMT) and pericyte-to-myofibroblast transition (PMT) (130). In EndMT, complement drives ECs to acquire a myofibroblast phenotype, contributing to vascular damage and early fibrosis (150), as demonstrated in preclinical models of AKI induced by LPS and I/R (151). Similarly, complement also promotes PMT and enhances renal fibrogenesis. The loss of pericytes, which play a key role in angiogenesis and vascular homeostasis, is a hallmark of AKI and correlates with the decline of kidney function (152–154). Another interesting aspect is the role of C3 and C1q complement components in macrophages polarization, an essential factor in AKI evolution. While classically activated M1 macrophages contribute to initial injury, conversion to M2 anti-inflammatory macrophages during the recovery phase is critical in resolving inflammation and restoring tubular function. Strikingly, their differentiation into the M1 or M2 phenotype is regulated by C3 and C1q (155, 156). Overall, available evidence supports a critical role of complement in accelerating the process of premature aging which characterizes AKI-CKD transition. This phenomenon is more marked in the elderly, increasing susceptibility to accumulate chronic irreversible lesions after AKI events (157).

### Potential Therapeutic Role of EVs in AKI-CKD Transition

Can EVs play a therapeutic role in reducing the risk of AKI-CKD transition? Although no study has specifically focused on this endpoint, there is some evidence from some of the previously mentioned studies that EV pleiotropic actions (**Table 1**) could inhibit AKI-CKD transition (51, 67). Activation of specific mediators such as Sox 9 (95) and transfer of miRNAs such as mi-R29b, which modulates Angiotensin 2-induced EMT of RTECs (158), are examples of effects which could be exploited to inhibit mechanisms leading to irreversible renal damage. Furthermore, as already mentioned, uEVs from healthy subjects can carry Klotho protein and transfer it to RTECs, restoring normal intra-tubular levels with beneficial effects on recovery from AKI (70).

Another interesting approach to prevent fibrosis after AKI is through complement blockade (159). A few studies suggest that EVs may exert an anti-complement activity through transfer of specific complement inhibitors. Cantaluppi et al. demonstrated that EPC-derived EVs could protect from complement mediated injury in experimental anti-Thy1.1 glomerulonephritis by transferring mRNAs coding for Factor H, CD55 and CD59 and related proteins to mesangial cell, thus inhibiting antibody/complement-induced apoptosis and C5b-9/C3 mesangial cell deposition (55). Similarly, EPC-derived EVs were able to preserve glomerular EC and podocyte integrity from complement-induced damage in a co-culture model mimicking the glomerular filtration barrier (160).

Although this setting is completely different from AKI, it is tempting to speculate that this mechanism of action might also explain some of the beneficial effects of EVs in AKI-CKD transition. Initial evidence indicates that human MSCs can ameliorate complement-induced inflammatory cascade and improve renal function at very early stages in experimental ischemic AKI, suggesting an immunomodulatory capacity possibly mediated by EVs (161). Finally, preliminary results from our group showed that EPC-derived EVs may limit ischemic AKI through complement inhibition (data not shown). Further studies are needed to investigate the potential of EVs as anti-complement therapy in order to prevent AKI-CKD transition. However, the potential of EV therapy to limit AKI development and AKI-CKD progression based on the horizontal transfer of proteins, receptors, bioactive lipids and different types of RNAs represents a great incentive for future research in this field.

## Limits and Perspectives of EV-Based Therapy

The use of SC-derived EVs as a therapeutic tool to deliver growth factors, proteins and genetic material to injured renal resident cells is promising in different AKI fields: however, several obstacles still limit their translation to clinic and have been recently reviewed (162, 163).

Biochemical composition is not defined and can vary depending on parental cell but also on surrounding milieu (e.g. inflammation, hypoxia). EVs released from the same cell-type may even have contradictory effects: for example, hypoxic RTECs have proved beneficial in alleviating tubular damage and fibrosis but injured RTECs can also release EVs which contribute to amplify inflammation (164). Moreover, focusing on SC-derived EVs, a different phenotype may depend on donor characteristics (autologous vs. heterologous, age, gender, presence of comorbidities or transient inflammatory states, etc.)

Furthermore, EV production or uptake mechanisms by kidney resident cells or infiltrating inflammatory cells are not completely defined and an intact glomerular filtration barrier could prevent EVs from reaching podocytes and tubular cells (165).

Finally, lack of good manufacturing practice standards and high-scale EV production hinder clinical application. At the moment, the clinical use of EVs is not classified as cell therapy and their mechanisms of action look like more to administration of a drug rather than a real cell therapy. Moreover, despite the development of new isolation procedures, cGMP production of SC-derived EVs for clinical application seems still to depend on an ultracentrifugation step to be performed within a cell factory.

Despite these limits, EV-based therapy has many strengths and is opening new therapeutic perspectives for a condition currently treated only with supportive therapy.

In general, EV lipid and surface protein composition (e.g.CD47) limits phagocytosis by circulating monocytes and prolongs blood half-life if compared to liposomes or other nanoparticles employed to carry drugs. EVs usually express integrins and adhesion molecules which allow to enhance their homing to inflamed or injured tissue: moreover, EVs protect RNA from degradation after their intravenous administration and at tissue level.

Technologies such as tangential flow filtration (TFF) appears to allow large-scale production of high-quality, reproducible EVs from AD-MSCs, paving the way for potential widespread clinical application in AKI (166). Other technological advances may potentiate EV qualitative therapeutic properties. For example, EV encapsulation could make their therapeutic content (e.g. miRNAs, mRNAs, proteins) more protected and stable. Nanomedicine techniques may help engineering EV features (size, shape, surface charge) in order to enable them to pass specific biological barriers, including glomeruli. Decoy exosomes have been proposed to antagonize inflammatory mediators (167). Another therapeutic approach is that of transfecting MSCs with specific miRNA mimics in order to enrich them with selected miRNAs. These enriched EVs proved to be more effective than naïve ones, potentially allowing the use of a lower amount of them (168). Combination therapy of pulsed focused ultrasound (pFUS) and EVs has proved more effective than either approach alone in reversing AKI-related inflammation through suppression of heat shock protein 70 – mediated NLPR3 inflammasome (169).

An interesting tool to increase therapeutic potential of MSCs is the adoption of three-dimensional (3D) culture of human placental MSC (hPMSCs), which proved to be more effective than two-dimension (2D) culture in preventing renal damage when injected in a mouse model of IRI-induced AKI (170). MSC 3D spheroid structures enable increased cell-cell interactions and enhance MSC trophic and immunomodulatory functions, with more reproducible clinical outcomes in many preclinical models (171) including cisplatin-induced AKI (172).

We have already discussed the potential of hypoxia and collagen matrix encapsulation to enhance EV protective effects, paving the way to new possibilities of therapeutic manipulation (106, 107, 116). Of note, the use of EVs avoid the possible adverse effects associated with whole cell therapies such as pulmonary embolism, vascular thrombosis, maldifferentiation and tumorigenesis (173).

## Conclusions

A growing body of evidence based on pre-clinical studies suggests that EVs released from MSCs of different origin and from other SC types could be effective to treat toxic, ischemic and septic AKI through direct interference with multiple etiopathogenetic mechanisms of tubular and endothelial damage. A network of cytoprotective, regenerative and immunomodulatory EV properties is being defined. EV-based therapy could prevent renal fibrosis and AKI-CKD transition, also through inhibition of complement-mediated processes such as EndMT and PMT. Hypoxia-conditioned and engineered EVs with enhanced therapeutic properties are promising new tools. Even though some technological hurdles must still be overcome before widespread clinical application, EV-based therapies may become a cornerstone for the treatment of the most common forms of AKI in the near future.

## Authors Contributions

MQ and VC designed and wrote the initial manuscript. GM and AC designed Figures (Created with BioRender.com) and Tables and arranged References. SB, AS, RF, EG, and VF reviewed the article focusing on experimental models. GC wrote the paragraph on AKI-CKD transition and all authors critically revised the whole article.

## Funding

This study was partially funded by the Italian Ministry of Education, University and Research (MIUR) program “Departments of Excellence 2018-2022”, AGING Project – Department of Translational Medicine, University of Piemonte Orientale (UPO) and by local grants of the University of Piemonte Orientale (UPO, FAR) to M.Q. and V.C.

## Conflict of Interest

The authors declare that the research was conducted in the absence of any commercial or financial relationships that could be construed as a potential conflict of interest.

## Publisher’s Note

All claims expressed in this article are solely those of the authors and do not necessarily represent those of their affiliated organizations, or those of the publisher, the editors and the reviewers. Any product that may be evaluated in this article, or claim that may be made by its manufacturer, is not guaranteed or endorsed by the publisher.

## Glossary

**Table:**

AKI	acute kidney injury
AD	adipose tissue
ATN	acute tubular necrosis
BM	bone marrow
CCL-2	chemokine ligand 2
CCR-2	chemokine receptor type 2
C1-INH	C1-Inhibitor
CKD	chronic kidney disease
CLP	cecal liagation and puncture
CM	contrast medium
DAMP	damage associated molecular pattern
DC	dendritic cell
EC	endothelial cell
ECFC	endothelial colony-forming cells
ECM	extracellular matrix
EMT	epithelial-to-mesenchimal transition
EndMT	endothelial-to-mesenchymal transition
EPC	endothelial progenitor cell
EV	extracellular vesicle
FGFR-1	fibroblast growth factor receptor 1
HGF	hepatocyte growth factor
HIF-1α	hypoxia-inducible factor 1
HLSCs	human liver stem cells
HO	heme-oxigenase
hP	human placenta
HPC	hypoxia pre-conditioning
ICU	intensive care unit
iPSC	induced pluripotent stem cells
IRI	ischemia-reperfusion injury
LPS	lipopolysaccharide
mDNA	mitochondrial DNA
miRNA	microRNA
MCP-1	monocyte chemoattractant protein 1
MSC	mesenchymal stromal cell
NADPH	nicotinamide adenine dinucleotide phosphate
NET	neutrophil extracellular trap
NOX	nicotinamide adenine dinucleotide phosphate oxidase
NRF-2	nuclear factor eryhroid 2-related factor 2
rIPC	remote ischemic pre-conditioning
PAMP	pathogen associated molecular pattern
PMN	polymorphonuclear cell
PMT	pericyte-to-mesenchymal transition
pFUS	pulsed focused ultrasound
ROS	reactive oxygen species
RRT	renal replacement therapy
RTEC	renal tubular epithelial cells
s-AKI	sepsis-associated AKI
SASP	senescence-associated secretory phenotype
SC	stem cell
TGFβ-1	Transforming Growth Factor β-1
TLR	Toll-like receptors
UC	umbilical cord
uEVs	urinary extracellular vesicles
VEGF	vascular endothelial growth factor
WJ	Warton Jelly
